# Genome-Wide Identification and Characterization of *14-3-3* Gene Family in Peanut

**DOI:** 10.3390/genes17080901

**Published:** 2026-07-30

**Authors:** Limei Li, Jie Peng, Gang Chen

**Affiliations:** Key Laboratory of Efficient Utilization of Special Biological and Medical Resources, School of Life Sciences, Zhaoqing University, Zhaoqing 526061, China

**Keywords:** *14-3-3* gene family, genome-wide identification, *Arachis hypogaea*, collinearity analysis, molecular characteristics

## Abstract

Background: Peanut (*Arachis hypogaea*) is a globally important legume crop with high nutritional and economic value. In the plant, the highly conserved 14-3-3 proteins participate in various biological functions. This study investigated the characterization and evolution of the *14-3-3* gene family in peanut and explores its potential roles in stress resistance. Methods: The *14-3-3* gene families in cultivated peanut and its ancestral species *Arachis duranensis* and *Arachis ipaensis* were identified and characterized via a genome-wide approach. We subsequently analyzed their structural features, conserved motifs, genomic distribution, phylogeny and promoter cis-acting elements. Results: A total of 22, 10, and 12 *14-3-3* genes (also called general regulatory factors, GRFs) were identified in *A. hypogaea*, *A. duranensis*, and *A. ipaensis*, respectively. Cultivated peanut exhibits extensive genomic synteny with its progenitors, and segmental duplication appears to be a key mechanism underlying the expansion of this gene family. Moreover, according to cis-acting element prediction, these genes may contribute to stress tolerance under diverse environmental conditions. Conclusion: This comprehensive analysis enhances our understanding of the *14-3-3* (*GRF*) gene family in peanut and provides a valuable basis for further functional research on these genes.

## 1. Introduction

The 14-3-3 proteins are soluble regulatory molecules first identified in bovine brain. Since their discovery, 14-3-3 proteins have been found in many other eukaryotes, including 13 members in Arabidopsis [1], 8 in rice [2], 17 in tobacco [3], 18 in soybean [4], 28 in maize [5], and 25 in banana [6]. In Arabidopsis, these proteins are also referred to as general regulatory factors (GRFs) [7,8]. The 14-3-3 protein family is highly conserved, and exists as homodimers or heterodimers in plants [3]. Despite their high sequence conservation, 14-3-3 isoforms perform distinct regulatory roles. They interact with target proteins through phosphorylation sites to regulate various cellular activities and physiological processes [9,10].

As core components of intracellular protein interaction networks, 14-3-3 proteins regulate signaling molecules in numerous signal transduction pathways. Moreover, due to their structural domains being able to simultaneously bind two different signaling molecules, they possess the function of connecting and coordinating different signal transduction pathways to form networks [11]. Different 14-3-3 isoforms exhibit distinct functional specificities, interacting with target proteins through the recognition of specific phosphorylated sequences. The binding of different isoforms may exert diverse effects on their targets, including conformational changes, subcellular relocalization, stability modulation, and altered protein–protein affinity [12]. For example, in guard cells, 14-3-3ε-GFP exhibited a predominantly cytoplasmic distribution with reduced nuclear presence. In contrast, 14-3-3κ-GFP showed an almost exclusively nuclear localization, indicating a striking difference in subcellular partitioning between these two highly similar isoforms [10]. Plant 14-3-3 proteins bind to target proteins via phosphorylation motifs and additionally function as scaffold proteins through interactions with target protein domains, thus participating in the regulation of a wide array of downstream targets.

In plants, 14-3-3 proteins play important roles in abiotic stress adaptation, including salinity, primarily through their interactions with and regulation of diverse targets. Under salt stress, 14-3-3 protein interacts with SOS3/SCaBP8 as calcium signal decoders, thereby regulating the downstream proteins SOS2 and PKS5 to maintain Na^+^ homeostasis [13]. In Arabidopsis roots, 14-3-3 proteins control salt-induced K^+^ efflux by binding to and activating CPK21, which controls the activity of the GORK channel [14]. Furthermore, the glutamate receptor-like protein GLR3.7 is involved in salt stress response in Arabidopsis through its modulation of calcium signaling, a process mediated by its interaction with the 14-3-3ω protein [15]. Similarly, GhN/AINV13 interacts with its positive regulator, the Gh14-3-3 protein, and this interaction facilitates its involvement in stress tolerance in cotton [16]. In rice, the OsGF14b protein positively regulates salt tolerance by interacting with and stabilizing OsPLC1, thereby enhancing both its activity and stability [17]. Collectively, these findings highlight that 14-3-3 proteins serve as central hubs in plant salt stress responses by interacting with diverse target proteins to regulate ion homeostasis, calcium signaling, and stress tolerance across different plant species [18].

Drought is a major factor limiting plant growth and development, and 14-3-3 proteins contribute broadly to plant adaption under such conditions. For instance, the 14-3-3 protein BdGF14a enhances the transcriptional regulatory activity of *BdbZIP62*, thereby conferring drought resistance through the ABA-mediated signaling pathway [19]. In Arabidopsis, AtGCN4 promotes drought tolerance by destabilizing the RIN4-14-3-3 complex, which reduces H^+^-ATPase activity and facilitates stomatal closure [20]. Additionally, overexpression of *GsGF14o* enhances ABA sensitivity and induces stomatal closure through interaction with AREB/ABF transcription factors [21]. Consistent with these findings, overexpression of *MdGRF11* upregulates stress-related gene expression and triggers both ABA signaling and ROS scavenging, collectively contributing to drought tolerance [22]. The 14-3-3 proteins serve as key integrators of ABA and ROS signaling. This functional centrality makes the 14-3-3 family particularly relevant for peanut, a crop that often encounters drought stress. These observations indicate that 14-3-3 proteins act as key regulators in plant drought responses by modulating transcription factor activity, stomatal movement, ABA signaling, and ROS homeostasis across different plant species.

Peanut is a major global oil crop that suffers substantial productivity losses due to abiotic stresses. Cultivated peanut (*Arachis hypogaea*) is a tetraploid species believed to have originated from two diploid ancestors, *Arachis duranensis* and *Arachis ipaensis* [23,24]. Polyploidization not only results in a genome merger but also triggers extensive genomic reorganization, such as transposable element activation, structural variations and epigenetic reprogramming, all of which collectively contribute to altered phenotypes and ecological adaptations [25]. The gene number does not simply double following polyploidization, as the genome undergoes reorganization, gene loss, and fractionation during the subsequent diploidization process. For gene families, expansion can also be driven by segmental duplication and whole-genome duplication (WGD) events, while gene loss and structural variations contribute to differences in gene family size among species.

Given that *14-3-3* genes regulate plant growth, development and adaptation to environmental stresses, a systematic characterization of the structure and function of the peanut *14-3-3* gene family is essential. These genes hold potential as molecular tools for developing peanut varieties with improved stress tolerance. To this end, this study presents a genome-scale characterization of the *14-3-3* gene family in peanut, including analyses of structural features, conserved motifs, genomic distribution, phylogeny, and promoter cis-acting elements. These findings provide a valuable reference for future investigations into the biological functions and agronomic potential of *14-3-3* genes in peanut.

## 2. Materials and Methods

### 2.1. A Genome-Wide Search for 14-3-3 Genes in Peanut

The genome sequences of *A. hypogaea* (cv. Tifrunner) and its corresponding gene annotations were obtained from the Ensembl Plants database (http://plants.ensembl.org/index.html, accessed on 7 January 2026). *A. duranensis* (cv. V14167) and *A. ipaensis* (cv. K30076) genome data were sourced from NCBI. To identify candidate 14-3-3 proteins, the 14-3-3 protein sequences of Arabidopsis were acquired from the TAIR database (https://www.arabidopsis.org/, accessed on 8 January 2026) and used as query sequences for BLASTp alignment. The search was performed using TBtools (Toolbox for Biologists) v2.376 with an E-value threshold of 1e-5 [26]. Subsequently, the candidate proteins were searched against the hidden Markov model (HMM) profile of the 14-3-3 domain (PF00244), which was obtained from the Pfam database (http://pfam-legacy.xfam.org/, accessed on 16 January 2026). Finally, all non-redundant peanut 14-3-3 protein sequences were validated using the NCBI CDD (http://www.ncbi.nlm.nih.gov/cdd/, accessed on 16 January 2026) and InterPro (http://prosite.expasy.org/, accessed on 16 January 2026) databases. According to chromosomal positions, all *14-3-3* genes in *A. hypogaea*, *A. duranensis*, and *A. ipaensis* were renamed *AhGRF1* to *AhGRF22*, *AdGRF1* to *AdGRF10*, and *AiGRF1* to *AiGRF12*, respectively.

### 2.2. Phylogenetic Tree Analysis

The Clustal W function in MEGA 11 was used to align the identified protein sequences of *A. hypogaea*, *A. duranensis*, and *A. ipaensis* along with the GRF sequences of Arabidopsis. A phylogenetic tree was subsequently constructed using the Maximum Likelihood (ML) method in MEGA11, with the Jones-Taylor-Thornton (JTT) model and 1000 bootstrap replicates. Bootstrap support values exceeding 50% were shown at the internal nodes. The resulting tree topology was visualized using the online platform Evolview (http://www.evolgenius.info/evolview/, accessed on 20 January 2026).

### 2.3. Characterizing Gene Structure, Conserved Domains, and Motifs

To understand the diversity of GRF family members, we analyzed their sequence features. The exon and intron arrangement of these genes were examined and displayed with TBtools v2.376 [26]. The same software was used to calculate the molecular weights and isoelectric points of the corresponding proteins. Conserved domains were identified through the NCBI CDD database. To further characterize the protein sequences, the MEME tool (https://meme-suite.org/meme/tools/meme, accessed on 20 January 2026) was employed to identify conserved motifs, with the maximum number of motifs set to 10. Finally, all identified domains and motifs were visualized using TBtools [26]. The subcellular distribution of the identified proteins was predicted by the Wolfpsort server (https://wolfpsort.hgc.jp/, accessed on 22 April 2026).

### 2.4. Chromosomal Location and Syntenic Analysis

To determine the chromosomal distribution and duplication patterns of the gene family, chromosomal location information of the predicted *GRF* genes was retrieved from the genomic sequences and annotation files. Duplication types and collinear blocks were identified using MCScanX in TBtools, which also generated a diagram of gene duplication events [26]. Duplication and divergence times were calculated using the formula described previously [23,27]. The MCScanX toolkit was used for syntenic analysis between *A. hypogaea* and its ancestors to examine the collinearity of *GRF* family genes, and the resulting figure was generated using TBtools [26].

### 2.5. Prediction of Promoter Cis-Acting Elements

To explore the potential transcriptional regulatory mechanisms, the upstream 2000 bp sequences immediately preceding the ATG start codon of all *AhGRF* genes were extracted from the *A. hypogaea* genomic sequences using TBtools [26]. These promoter sequences were screened against the PlantCARE database (https://bioinformatics.psb.ugent.be/webtools/plantcare/html/, accessed on 20 January 2026) to predict cis-acting elements. Finally, a schematic diagram illustrating the element distribution on each promoter was generated using TBtools [26].

### 2.6. Expression Analysis of AhGRF Genes

To investigate the transcriptional profiles of *AhGRF* genes, their expression was examined using RNA-seq datasets from the PeanutBase database (https://data.legumeinfo.org/Arachis/hypogaea/expression/, accessed on 23 July 2026). The RNA-seq data analyzed in this study were not generated as part of this work but were obtained from a previously published transcriptomic study [28]. Two-week-old Yueyou 7 peanut seedlings were treated with 100 µM abscisic acid (ABA) for 30 min, followed by incubation in 30% PEG 6000 solution for 30 min. Untreated seedlings maintained under identical conditions served as the control group. Each treatment was performed with three biological replicates. After treatments, total RNA was extracted from 100 mg of leaf tissue and then subjected to RNA-seq [28]. The raw sequencing reads and associated metadata for this study are publicly available under the NCBI BioProject accession PRJNA243319. The TPM values were retrieved from the PeanutBase database (https://data.legumeinfo.org/Arachis/hypogaea/expression/Tifrunner.gnm2.ann2.expr.Tifrunner.Li_Lu_2014/) (Table S1), and the heatmap was constructed using log2(TPM+1) transformation followed by row-wise Z-score normalization in TBtools [26].

## 3. Results

### 3.1. Identification of 14-3-3 Family Members in Peanut

Based on the 14-3-3 protein sequences of Arabidopsis from the TAIR database, BLAST and HMM searches were executed against the genome of cultivated tetraploid peanut (*A. hypogaea*) to identify members of the 14-3-3 family using TBtools. Following domain verification using the NCBI CDD and InterPro databases, a total of 22 *14-3-3* genes were identified in the *A. hypogaea* genome and designated *AhGRF1* to *AhGRF22*, according to their order on chromosomes. The AhGRF proteins are characterized by small molecular weights, ranging from 16.90 kDa to 33.59 kDa, and predicted isoelectric points (pI) between 6.67 and 8.92 (Table 1). These proteins were predicted to localize to the nucleus, chloroplast, cytoplasm, mitochondria and plasma membrane (Table 1). This study provides the first comprehensive genome-wide identification and characterization of the *GRF* family in cultivated peanut.

To elucidate the differences in 14-3-3 family members between cultivated tetraploid peanut and its wild diploid progenitors, the 14-3-3 protein sequences in *A. duranensis* and *A. ipaensis* were also identified using identical methods. Finally, 10 members were identified in *A. duranensis* and 12 in *A. ipaensis*, which were named *AdGRF1* to *AdGRF10* and *AiGRF1* to *AiGRF12*, respectively. For the diploid species, AdGRF proteins consist of 252 to 295 amino acids, exhibiting molecular weights between 28.12 kDa and 34.13 kDa and isoelectric points between 4.67 and 5.09. AiGRF proteins range from 147 to 276 amino acids, with molecular weights varying from 17.32 kDa to 31.49 kDa and isoelectric points from 4.67 to 8.41 (Table S2). The physicochemical properties of GRF proteins in diploid species are distinctly different from those in tetraploid peanut. Taken together, regarding the physicochemical properties of the sequences, the GRF sequences diverged during the transition from diploid to tetraploid, although the sum of gene numbers in the two diploids equals that in the tetraploid.

### 3.2. Phylogenetic Relationships Among GRF Family Proteins

**Figure 1:**
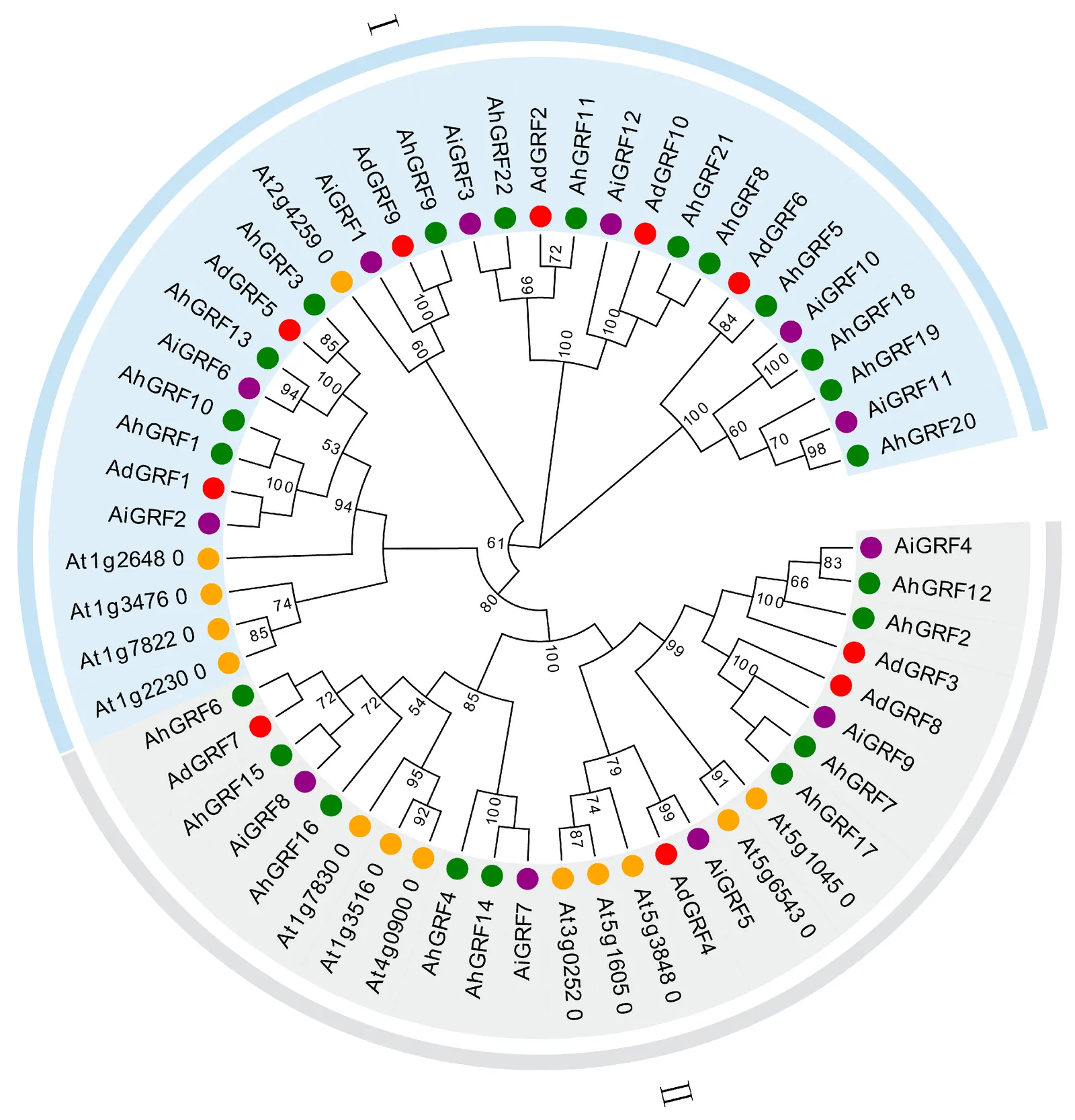
Phylogenetic tree of GRF proteins from *A. hypogaea*, *A. duranensis*, *A. ipaensis* and Arabidopsis. Protein sequences were aligned by the Clustal W function in MEGA 11, and the phylogenetic tree was constructed with the Maximum Likelihood (ML) method. Colors distinguish group I from group II. Green, red, purple and yellow circles denote proteins from *A. hypogaea*, *A. duranensis*, *A. ipaensis* and Arabidopsis, respectively.

To understand the phylogeny of GRF family proteins, we performed phylogenetic analyses on 22 AhGRFs from *A. hypogaea*, 10 from *A. duranensis*, 12 from *A. ipaensis*, and 13 from Arabidopsis. A total of 57 GRF protein sequences were aligned, and a Maximum Likelihood (ML) tree was subsequently generated. Phylogenetic analysis divided all GRFs into two groups: group I contained 31 members, and group II contained 26 members (Figure 1). The phylogenetic relationships were further examined using the (Neighbor-joining) NJ method in MEGA 11 (Figure S1). The NJ tree was largely consistent with the ML tree in terms of overall topology, especially for the major subfamily groups, supporting the robustness of our phylogenetic classification. Members within the same group generally share similar conserved motif compositions and gene structures, suggesting that they may have evolved from common ancestors and potentially exhibit functional redundancy or cooperative roles.

### 3.3. Analysis of GRF Structural Features and Conserved Protein Motifs

**Figure 2:**
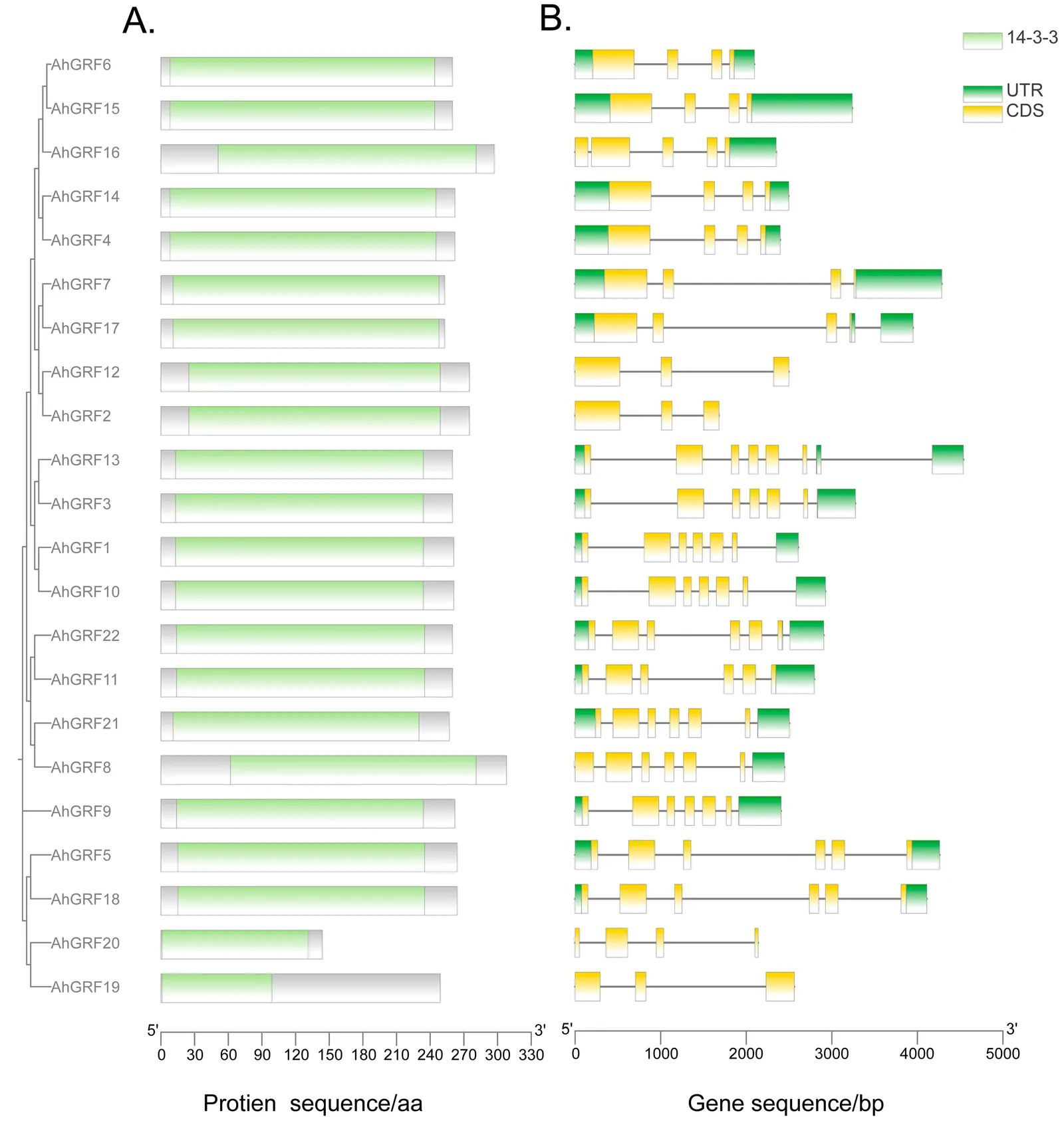
Structural features of *AhGRF* family in *A. hypogaea*. (**A**) The conserved domains of AhGRF proteins. (**B**) The gene structures of *AhGRF* genes.

Structural analysis is essential for revealing the evolutionary conservation and functional diversity of the GRF family in peanut. To investigate the structural features of *GRF* family members, conserved domains and gene structures were analyzed. NCBI CDD database analysis confirmed that all identified GRF protein sequences contain the 14-3-3 domain (Figure 2A). The gene structure of *AhGRFs* consists of variable numbers of exons and introns. Across the 22 *AhGRF* genes, the number of introns varies, and all genes contain three to six exons, with most members possessing six exons. In addition, the length and structural organization of the untranslated regions (3′ UTR and 5′ UTR) are highly variable. (Figure 2B). Overall, gene structure analysis revealed that although the structures of introns and UTRs vary considerably, the essential coding sequences remain conserved across all *GRF* family genes in *A. hypogaea*.

In *A. duranensis* and *A. ipaensis*, gene structure mapping revealed that all *GRF* genes are composed of introns and exons. The exon count varies from 4 to 7 in *A. duranensis* and from 4 to 6 in *A. ipaensis*. Notably, the majority of *GRF* genes in both species contain six exons (Figures S2 and S3). Based on conserved domain and gene structure analyses, *GRF* family members in peanut exhibit high similarity, indicating that most *GRF* genes are highly conserved.

**Figure 3:**
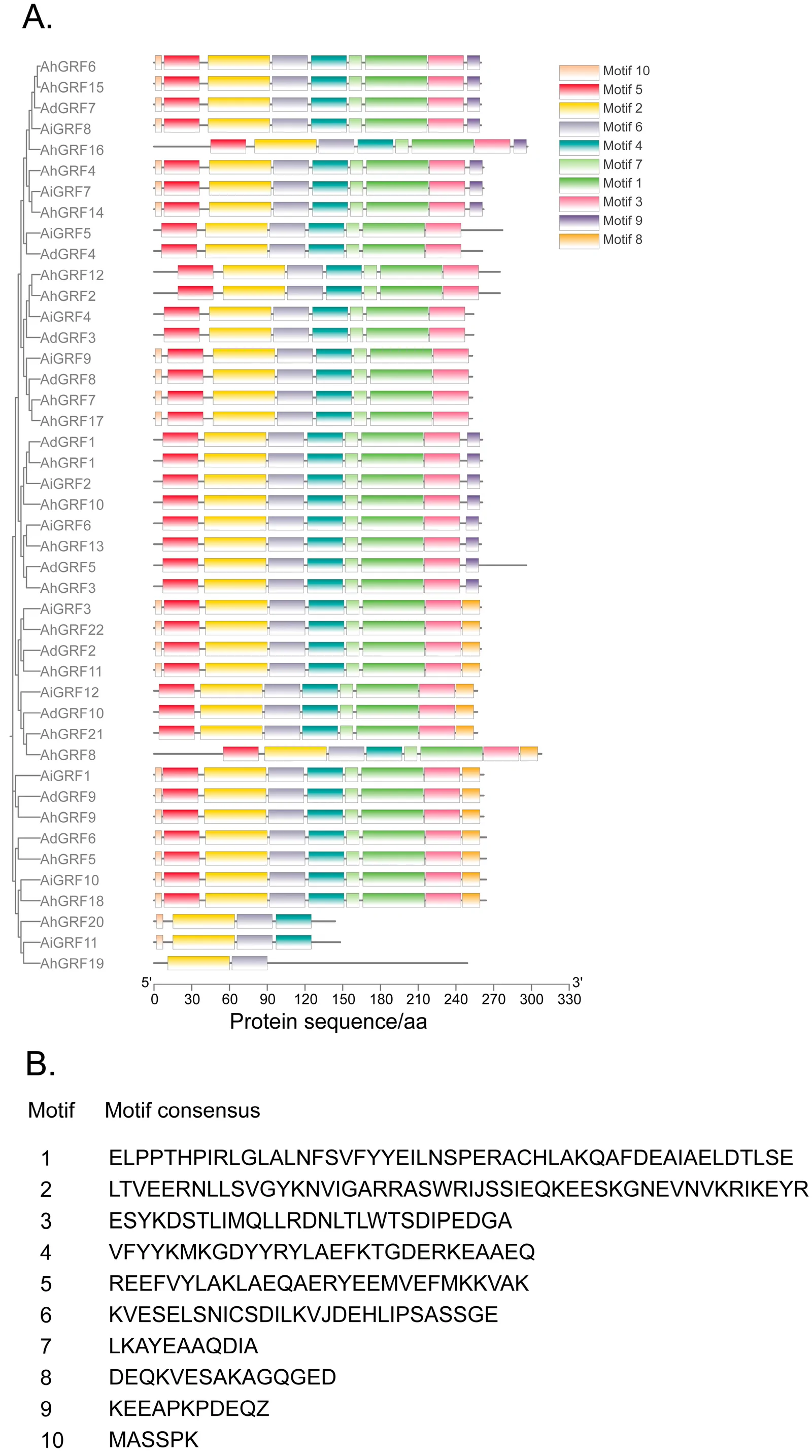
Conserved motifs of GRF proteins in *A. hypogaea*, *A. duranensis*, and *A. ipaensis*. (**A**) The conserved motifs of GRFs. A total of ten conserved motifs are distinguished by boxes with distinct colors. (**B**) The amino acid composition of each motif.

To identify conserved motifs of GRF protein sequences in *A. hypogaea*, *A. duranensis*, and *A. ipaensis*, we analyzed all sequences using the online tool MEME. Ten conserved motifs (Motif 1 to Motif 10) were found. Proteins are arranged following the phylogenetic tree order, and the conserved motif patterns are largely consistent with the phylogenetic grouping. These motifs range in length from 6 to 50 amino acids. The number of motifs varies from 2 to 9, with all members containing both Motif 2 and Motif 6, indicating that these two motifs are conserved across peanut GRF proteins. The presence of these conserved motifs suggests that they may be functionally important for 14-3-3 activity. The motifs are present in most family members and are arranged in the following order: Motif 5, Motif 2, Motif 6, Motif 4, Motif 7, Motif 1, Motif 3. In contrast to other family members, AhGRF20, AiGRF11, and AhGRF19 contain fewer motifs. AhGRF20 and AiGRF11 possess only four motifs (Motif 10, Motif 2, Motif 6, and Motif 4), whereas AhGRF19 contains only Motif 2 and Motif 6 (Figure 3). These reductions may be attributed to mutations during evolution and could be associated with their specialized biological functions. These genes may serve as promising candidates for future functional characterization.

### 3.4. Chromosomal Localization and Collinearity Patterns of AhGRF Genes

**Figure 4:**
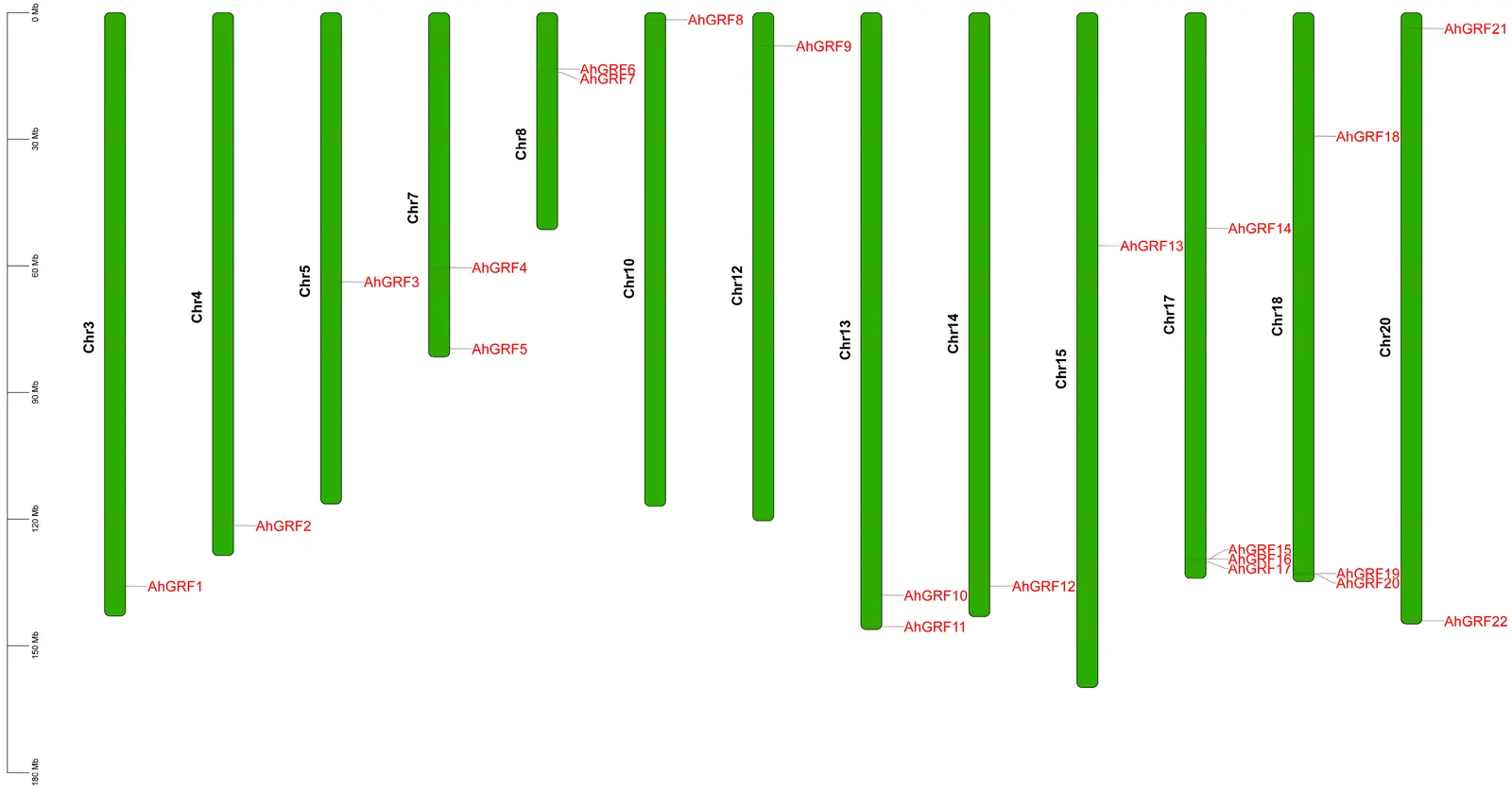
Distribution of *AhGRF* family genes on *A. hypogaea* chromosomes.

The chromosomal locations of all identified *AhGRF* genes in *A. hypogaea* were determined based on their physical positions within the genome. Chromosomal mapping showed that *AhGRF* genes are organized into three clusters on distinct chromosomes: *AhGRF6*-*AhGRF7*, *AhGRF15*-*AhGRF17*, and *AhGRF19*-*AhGRF20*. Apart from these clustered regions, the remaining *AhGRF* genes are loosely distributed across the chromosomes, with most located individually on separate chromosomes. However, several chromosomes, namely Chr1, Chr2, Chr6, Chr9, Chr11, Chr16, and Chr19, contain no *AhGRF* genes (Figure 4).

**Figure 5:**
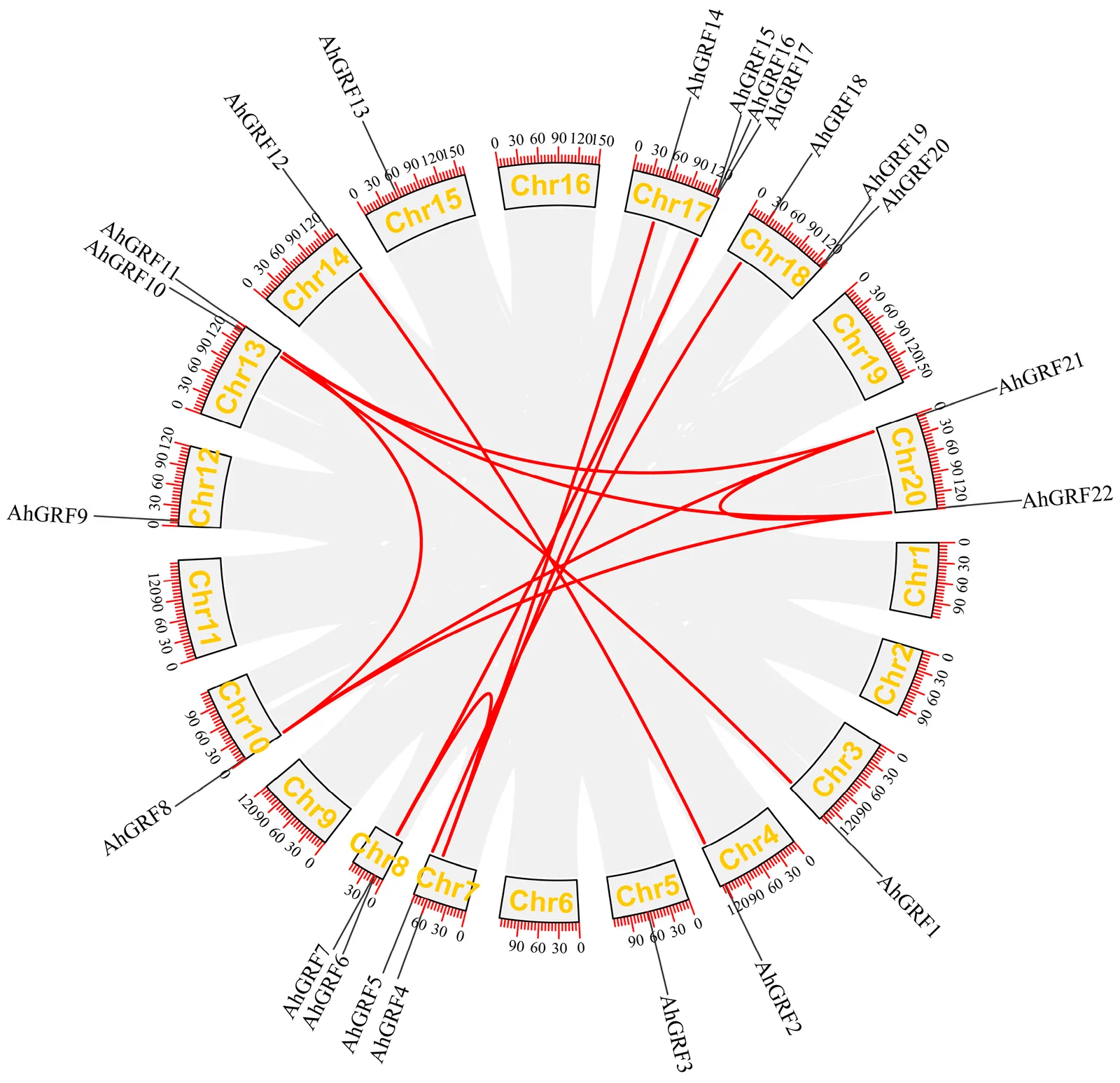
Circos plot of *AhGRF* duplication links.

Collinearity analysis revealed that 14 pairs of segmentally duplicated genes were identified among the 22 *AhGRF* genes (Figure 5). Specifically, *AhGRF1* on chromosome 3 paired with *AhGRF10* on chromosome 13. *AhGRF2* on chromosome 4 paired with *AhGRF12* on chromosome 14. *AhGRF4* on chromosome 7 paired with *AhGRF6* on chromosome 8, as well as with *AhGRF14* and *AhGRF15* on chromosome 17. *AhGRF5* on chromosome 7 paired with *AhGRF18* on chromosome 18, and *AhGRF21* on chromosome 20 paired with *AhGRF22* on chromosome 20. *AhGRF6* and *AhGRF7* on chromosome 8 paired with *AhGRF15* and *AhGRF17* on chromosome 17, respectively. *AhGRF8* on chromosome 10 paired with *AhGRF11* on chromosome 13, as well as with *AhGRF21* and *AhGRF22* on chromosome 20. *AhGRF11* also paired with *AhGRF21* and *AhGRF22*. In contrast, *AhGRF3*, *AhGRF13*, *AhGRF16*, *AhGRF19*, and *AhGRF20*, located on chromosomes 5, 15, 17, 18, and 18 respectively, were found to have no collinear pairings (Table S3). The lack of collinearity for these *AhGRF* genes suggests they may have undergone distinct evolutionary trajectories compared with their syntenic counterparts in related genomes. Whether this genomic divergence is accompanied by functional neofunctionalization remains unclear and would require further experimental investigation.

The ratio of nonsynonymous substitution rate to synonymous substitution ratesubstitutions (Ka/Ks) was determined for *AhGRF* gene pairs. A Ka/Ks value below 1 is evidence of purifying selection, while a value exceeding 1 points to positive selection. The Ka/Ks ratios for the *AhGRF* gene pairs ranged from 0 to 0.517, all of which are below 1, suggesting that these gene pairs were strongly subjected to purifying selection (Table S3). The segmental duplication events were estimated to have occurred approximately 0.725 to 92.2 million years ago (mya).

### 3.5. Evolutionary Syntenic Relationship Analysis of AhGRF Genes

**Figure 6:**
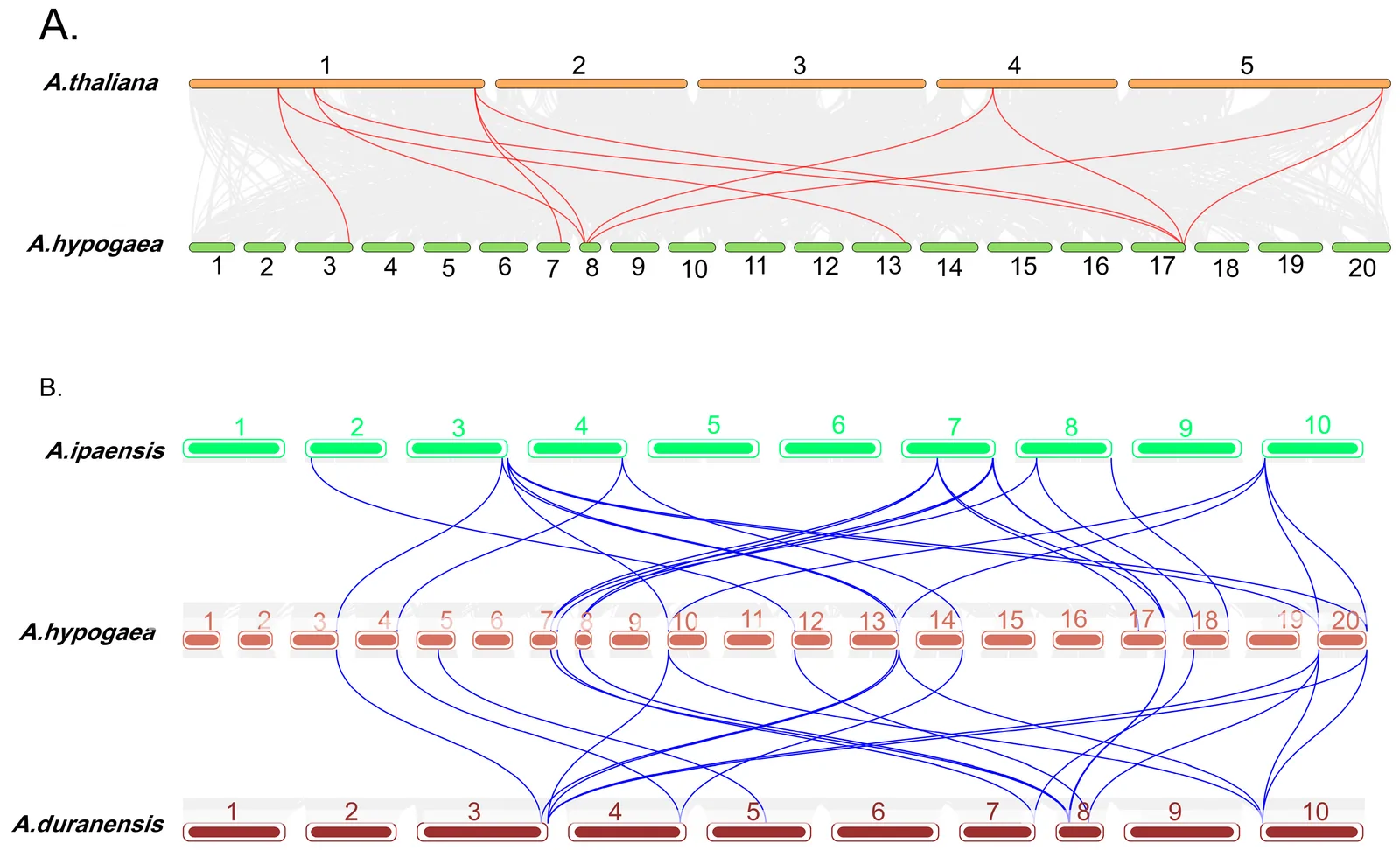
Genome-wide syntenic analysis of *GRF* genes. (**A**) Syntenic analysis of *GRF* genes between Arabidopsis and *A. hypogaea*. (**B**) Syntenic analysis of *GRF* genes between *A. hypogaea* and its two diploid ancestors, *A. duranensis* and *A. ipaensis*.

Arabidopsis was chosen as a reference due to its well-established phylogenetic position within the angiosperms, its complete and high-quality genome assembly, and the extensive functional studies available for its GRF family, which together facilitate the identification and classification of homologous genes in peanut. To gain insight into the evolutionary history of the *GRF* gene family, a genome-wide comparison of syntenic blocks was performed between Arabidopsis and *A. hypogaea* (Figure 6A). A total of 11 collinear *GRF* gene pairs were detected between these two species (Table S4). These 11 pairs were distributed among five *GRF* genes from Arabidopsis and five *GRF* genes from *A. hypogaea*, suggesting that these gene pairs are derived from a common ancestral origin. The orthologous relationships between peanut and Arabidopsis *GRF* genes were not uniform: some were identified as one-to-one orthologs, whereas others showed multiple-to-multiple correspondences.

To clarify the expansion and clustering patterns of the *GRF* gene family, we performed syntenic relationship analyses between *A*. *hypogaea* and its two wild diploid ancestors, *A. duranensis* and *A. ipaensis* (Figure 6B). These analyses provided valuable insights into the evolutionary relationships of *GRF* genes among multiple peanut species. Collinearity analysis further revealed 22 collinear loci between *A. hypogaea* and *A. ipaensis*, and 20 collinear loci between *A. hypogaea* and *A. duranensis* (Table S5). Collectively, these findings demonstrate that the *GRF* gene family remained evolutionarily conserved during the polyploidization event in peanut and enhance the reliability of the gene set, providing a solid foundation for downstream functional characterization in peanut.

### 3.6. Prediction of Cis-Acting Regulatory Elements in AhGRF Genes

**Figure 7:**
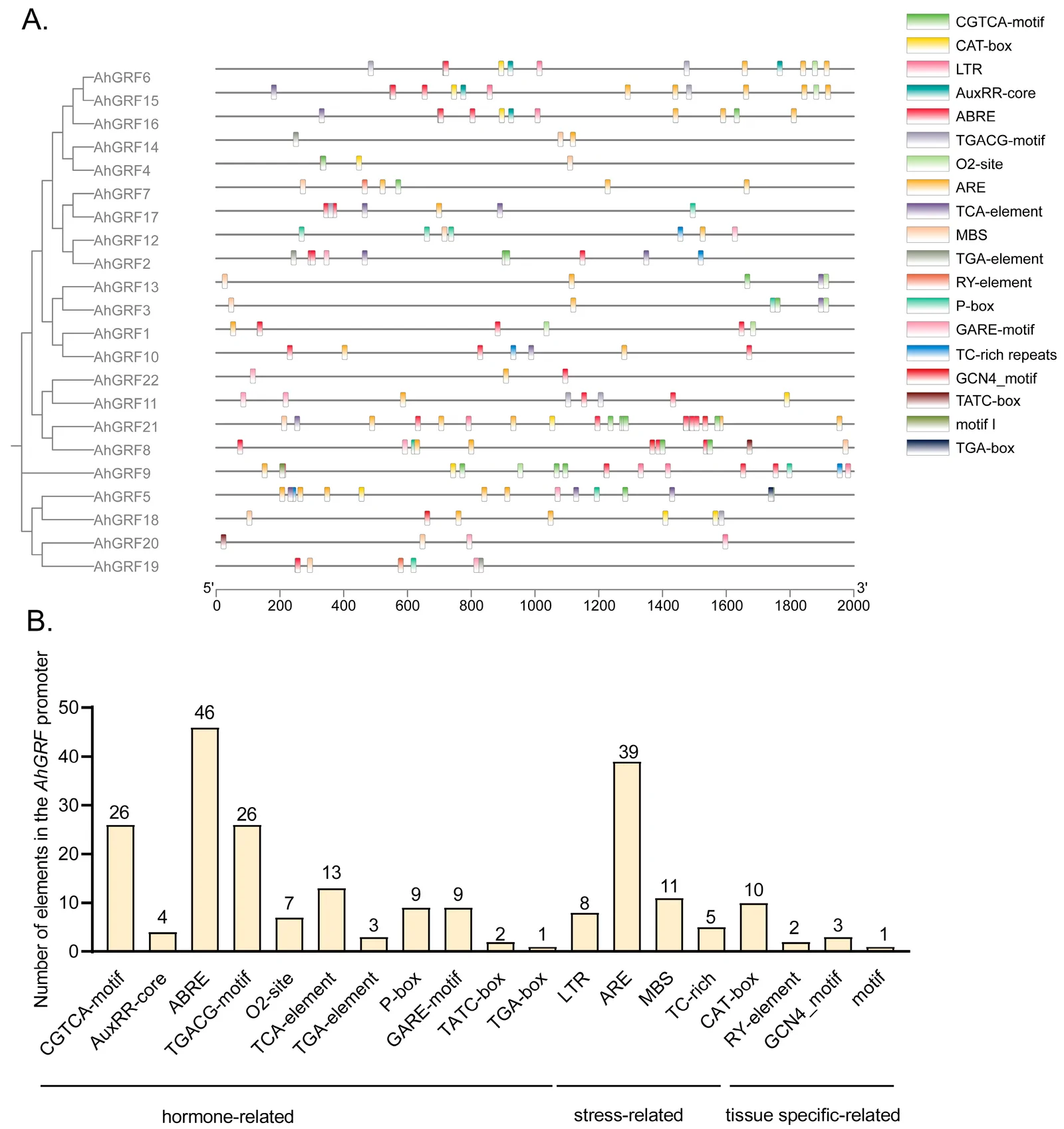
Cis-acting elements in *AhGRF* promoters of *A. hypogaea*. (**A**) Different types of elements are distinguished by colored boxes. (**B**) Number of cis-acting elements in the promoter of *AhGRF*.

To investigate the potential regulatory framework of *AhGRF* genes, the 2000 bp upstream sequences of the translation start site were extracted for cis-acting element identification (Figure 7). Hormone-responsive elements were widely distributed among *AhGRF* promoters. Specifically, MeJA-responsive elements were found in 15 members, suggesting a potential role in methyl jasmonate signaling. The ABRE motif (abscisic acid-responsive element) was detected in 14 members. The predominance of MeJA- and ABA-responsive cis-acting elements in the promoter regions suggests that *AhGRF* genes are potentially important for stress signal transduction. Auxin-responsive elements (AuxRR-core, TGA-element, and TGA-box), salicylic acid-responsive elements (TCA-element), and gibberellin-responsive elements (TATC-box) were found in 7, 9, and 2 members, respectively. In addition to hormone-related elements, cis-acting elements associated with abiotic stress were also identified. The TC-rich repeats, associated with defense and stress responsiveness, were found in *AhGRF2*, *AhGRF5*, *AhGRF9*, *AhGRF10*, and *AhGRF12*. ARE, an anaerobic induction element, was present in the promoters of the majority of members (18 out of 22). Furthermore, MBS (drought-inducibility) and LTR (low-temperature responsiveness) were found in 11 and 6 members, respectively. Together, these findings demonstrate that *AhGRFs* may play regulatory roles in stress response and tolerance signaling pathways. The diversity of hormone- and stress-responsive elements implies that these genes are subject to complex, multi-layered transcriptional regulation under stress conditions, thereby contributing to environmental adaptation in peanut.

Tissue-specific elements were also detected in several *AhGRF* gene promoters. A root-specific element (motif I) was detected exclusively in the promoter region of *AhGRF9*. A seed-specific regulatory element (RY-element) was present in *AhGRF7* and *AhGRF19*. An endosperm expression element (GCN4-motif) was found in *AhGRF8*, *AhGRF18*, and *AhGRF21*. Furthermore, the CAT-box, associated with meristem expression, was identified in nine family members. The distribution of these tissue-specific elements implies distinct developmental roles for *AhGRF* genes in peanut.

### 3.7. Expression Profile of AhGRF Genes

**Figure 8:**
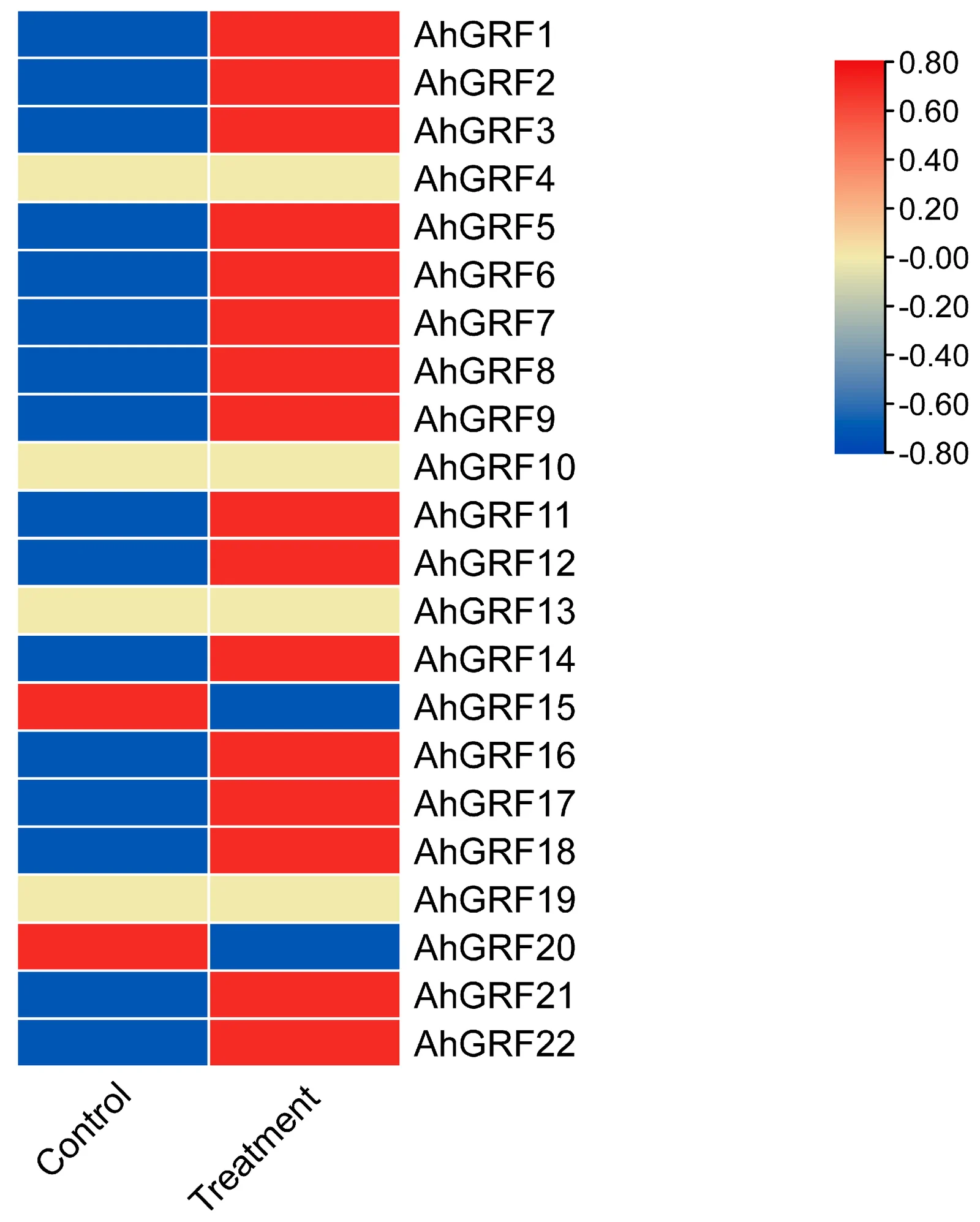
Expression profile of *GRF* family *in A. hypogaea*. Control: no treatment. Treatment: peanut seedlings were subjected to combined drought and ABA treatment, consisting of exposure to 100 µM ABA for 30 min, followed by incubation in 30% PEG 6000 solution for 30 min.

Promoter analysis revealed abundant hormone- and stress-related cis-acting elements in *AhGRF* promoters. To validate this, we examined *AhGRF* expression patterns using transcriptomic data from peanuts subjected to combined drought and ABA treatment. Most *AhGRF* genes were upregulated under this treatment (Figure 8), implying their potential roles in ABA-dependent stress signaling in peanut.

## 4. Discussion

As a conserved gene family, *14-3-3* genes are present in various plant species and contribute to a wide range of biological functions. In particular, these proteins play pivotal roles in regulating key rate-limiting enzymes in plant metabolism (e.g., sucrose phosphate synthase and nitrate reductase), mediating phytohormone signaling (e.g., GA and ABA pathways), and controlling cell cycle progression, nutrient metabolism, and stress resistance [18,29,30,31,32]. This conservation likely underpins the functional versatility of 14-3-3 proteins, enabling their participation in diverse physiological processes in plants. Functional studies have further elucidated their involvement in various stress responses. For instance, OsGF14d functions as a central regulator of rice chilling tolerance, with its phosphorylation status controlled by LENG via modulation of OsCRPK1 kinase activity [33]. In apple, MhSHINE2-like and MhGRF3 co-regulate downstream gene expression, leading to ABA accumulation and enhanced drought tolerance [34]. In pepper, CaTFT7 positively regulates resistance to bacterial wilt by stabilizing CaHDZ27 and enhancing its transcriptional activation of the defense-related gene *CaCRK5* [35]. Additionally, SINAT-mediated proteolysis of ATG6 is controlled by 14-3-3 proteins in Arabidopsis [36]. This finding exemplifies how 14-3-3 proteins integrate with the ubiquitin-proteasome pathway to modulate stress-related signaling in plants. Although 14-3-3 proteins have been extensively studied in many plant species, their characterization in peanut remains limited, highlighting the need for further investigation. Given the economic importance of peanut as a major oilseed and food crop worldwide, understanding the molecular mechanisms that govern its stress tolerance is essential for genetic improvement and sustainable production.

Here, we systematically identified and characterized the *14-3-3* gene family across the peanut genome. A total of 22, 10, and 12 *14-3-3* genes were identified in *A. hypogaea*, *A. duranensis*, and *A. ipaensis*, which were designated as *AhGRFs*, *AdGRFs*, and *AiGRFs*, respectively, to maintain consistent nomenclature across species. The number of *AhGRF* genes (22) in *A. hypogaea* is higher than that reported in Arabidopsis (13) and soybean (18). This difference may be explained by the recent polyploidization event in peanut, which allowed for the retention of most duplicated gene copies. By contrast, the older polyploidization events in Arabidopsis and soybean were followed by extensive gene loss during polyploidization. Notably, the total number of *GRF* genes in the two diploid progenitors (*A. duranensis* and *A. ipaensis*) is exactly equal to that in the tetraploid peanut *A. hypogaea*. However, this observation does not preclude the occurrence of sequence variations during the evolutionary process. In addition to the increase in gene number, structural divergence among GRF genes may underpin functional diversification. Therefore, to comprehensively characterize the *GRF* gene family, we analyzed their sequences features, protein properties, subcellular localization, phylogeny, synteny, and cis-acting regulatory elements using relevant databases and software tools. Furthermore, the interrelationships among *A. hypogaea* and its diploid progenitors were also investigated.

The functional importance and diversity of GRF proteins correspond to their broad subcellular localization. Here, we predicted the subcellular localization of GRF proteins in peanut and observed a broad distribution. In *A. duranensis* and *A. ipaensis*, GRF proteins were found to localize to the cytoplasm, plasma membrane, nucleus and chloroplasts. Similarly, most GRF proteins in *A. hypogaea* exhibited the same localization pattern, with the exception of AhGRF16. Specifically, AhGRF16 is predicted to localize to the mitochondria. These results indicate that the subcellular localization of GRF proteins has undergone changes during peanut polyploidization, which may consequently affect their biological functions.

Mitochondrial ATP synthase plays a central role in plant metabolism by converting the electrochemical proton gradient into ATP. Evidence from a previous study indicates that ATP synthase is regulated by 14-3-3 proteins [37]. Localizing to the inner mitochondrial membrane, 14-3-3 proteins interact with ATP synthase via phosphorylation to modulate its activity [37]. This 14-3-3-mediated regulation of ATP synthase represents a key mechanism underlying plant adaptation to changing environmental conditions. The mitochondrial localization of AhGRF16 implies that it may be involved in energy metabolism and stress adaptation, consistent with the known functions of mitochondria in plant stress responses. Whether AhGRF16 proteins in peanut participate in this regulatory mechanism remains an open question for future study. Nonetheless, this observation underscores the potential functional significance of 14-3-3 proteins in peanut.

Phylogenetic analysis uncovers the molecular and evolutionary basis of lineage relationships, and diversity among species. Based on the phylogenetic tree of GRFs from *A. hypogaea*, *A. duranensis*, *A. ipaensis*, and Arabidopsis, the GRFs were classified into two groups. Consistently, conserved motif analysis revealed that the most closely related members within each group shared similar protein structures and motifs. However, the motif configuration and number in AhGRF19, AhGRF20 and AiGRF11 differed from those of other proteins, exhibiting their distinct genetic feature and divergence in evolution. Furthermore, gene structure, defined by the configuration of exons and introns, simplifies the assessment of structural diversity within gene families. Here, most *GRF* genes were found to contain six exons, a feature that is conserved across different peanut species. In contrast, the number of introns in peanut *GRFs* is highly variable. Variation in intron number and positioning is generally considered a common evolutionary process [38]. Interestingly, studies have suggested that genes with more and longer introns tend to exhibit higher expression levels and can be activated more rapidly than those with fewer introns [39].

Cultivated peanut (*A. hypogaea*) is a tetraploid species originating from the diploid progenitors *A. duranensis* and *A. ipaensis* [23]. Syntenic analysis involves examining genomic fragments that originate from a common ancestor across different species. Using this approach, 22 syntenic gene pairs were identified between *A. hypogaea* and *A. ipaensis*, and 20 between *A. hypogaea* and *A. duranensis*. Segmental, tandem, and transposition duplications serve as key drivers of gene family expansion across a broad range of species [40]. These duplication events generate genetic variation, which may facilitate the emergence of novel gene functions or adaptations [41]. Consequently, gene duplication drives both plant gene family evolution and stress response capacity. Among the various types of duplications, tandem duplications refer to two or more genes located adjacently within the same chromosome, while segmental duplications involve duplicated genes situated on different chromosomes as a result of larger-scale chromosomal rearrangements [42]. Both types have contributed significantly to gene family expansion and the creation of genetic diversity in plants. In our analyses, 14 *AhGRF* gene pairs were classified as segmental duplication genes, suggesting that segmental duplication drove *GRF* family expansion after polyploidization in cultivated peanut.

The 14-3-3 protein family is known to participate in hormone signaling and stress tolerance [18,30]. There is growing evidence that 14-3-3 proteins can synergize with brassinosteroids (BRs), abscisic acid (ABA), gibberellin (GA) and auxin [43,44,45,46]. The ability of 14-3-3 proteins to interact with multiple hormone signaling pathways reflects their role as central hub proteins in plant signaling networks, coordinating diverse physiological outputs in response to developmental and environmental cues. This functional versatility is largely attributed to transcriptional regulation, where the promoter plays a critical role. Cis-acting elements within the promoter bind specific transcription factors to regulate the level, location, and timing of gene expression, constituting a key mechanism controlling gene expression [47]. In this study, Numerous stress- and hormone-related cis-acting elements were found in *AhGRF* promoters through prediction analysis. In addition to the promoter analysis, transcriptomic data also revealed that most *AhGRF* genes are responsive to drought and ABA, further supporting their potential involvement in ABA-mediated stress signaling. Among the identified cis-acting elements, MeJA-responsive and ABA-responsive elements were particularly predominant, suggesting that these elements may serve as major drivers of stress-responsive regulation in *AhGRF* genes. This observation aligns with known stress adaptation pathways previously documented in legumes, reinforcing the potential role of *GRF* family members in peanut stress responses.

## 5. Conclusions

In summary, a total of 22, 10, and 12 *GRF* genes were identified in *A. hypogaea*, *A. duranensis*, and *A. ipaensis*, respectively. Based on systematic analyses of their classification, structural features, conserved motifs, cis-acting elements, genomic distribution and phylogeny, this study establishes a basis for further exploration of the molecular and functional characteristics of *AhGRFs*. Specifically, our results revealed evolutionary conservation between *A. hypogaea* and its ancestral species *A. duranensis* and *A. ipaensis*. This comprehensive analysis enhances our understanding of the *GRF* gene family in peanut and provides valuable resources for further functional studies and genetic improvement in stress tolerance in cultivated peanut.

## Figures and Tables

**Table 1:** *AhGRF* family members identified in the *A. hypogaea* genome.

Gene Name	Gene ID	Amino Acids	MW/kDa	pI	Localization
AhGRF1	Ah03g531000	260	29.75	4.81	Nucleus
AhGRF2	Ah04g388600	274	30.56	4.99	Chloroplast
AhGRF3	Ah05g249000	259	29.60	4.84	Cytoplasm
AhGRF4	Ah07g253100	261	29.31	4.72	Nucleus
AhGRF5	Ah07g329000	263	30.07	4.79	Chloroplast
AhGRF6	Ah08g093500	259	29.20	4.67	Plasma membrane
AhGRF7	Ah08g098500	252	28.50	4.74	Chloroplast
AhGRF8	Ah10g020300	307	34.85	5.2	Plasma membrane
AhGRF9	Ah12g063800	261	29.48	4.81	Chloroplast
AhGRF10	Ah13g559900	260	29.78	4.81	Nucleus
AhGRF11	Ah13g640100	259	29.28	4.79	Chloroplast
AhGRF12	Ah14g457100	274	30.71	5.34	Nucleus
AhGRF13	Ah15g246800	259	29.62	4.87	Chloroplast
AhGRF14	Ah17g257800	261	29.35	4.72	Nucleus
AhGRF15	Ah17g464700	259	29.20	4.67	Plasma membrane
AhGRF16	Ah17g464800	296	33.59	5.12	Mitochondria
AhGRF17	Ah17g471700	252	28.50	4.74	Chloroplast
AhGRF18	Ah18g201700	263	30.03	4.8	Chloroplast
AhGRF19	Ah18g459000	248	28.57	8.92	Nucleus
AhGRF20	Ah18g460300	143	16.90	7.74	Nucleus
AhGRF21	Ah20g041400	256	28.92	4.83	Plasma membrane
AhGRF22	Ah20g526500	259	29.27	4.79	Chloroplast

## Data Availability

The datasets generated during the current study are available from the corresponding author upon reasonable request.

## Supplementary Materials

The following supporting information can be downloaded at: https://www.mdpi.com/article/10.3390/genes17080901/s1, Figure S1: Neighbor-joining (NJ) phylogenetic tree of GRF proteins from Arabidopsis, *A. hypogaea*, *A. duranensis*, and *A. ipaensis*. Protein sequences were aligned using the Clustal W function in MEGA 11, and the tree was constructed with the NJ method based on 1000 bootstrap replicates. Colors distinguish group I from group II. Green, red, purple, and yellow circles denote proteins from *A. hypogaea*, *A. duranensis*, *A. ipaensis*, and Arabidopsis, respectively. Bootstrap support values exceeding 50% are shown at the internal nodes. The resulting topology was visualized using Evolview. Phylogenetic analysis divided all GRFs into two groups: group I contained 31 members, and group II contained 26 members. Figure S2: Structural features of *AdGRF* family in *A. duranensis*. (**A**) The conserved domains of AdGRF proteins. (**B**) The gene structures of *AdGRF* genes. Figure S3: Structural features of *AiGRF* family in *A. ipaensis*. (**A**) The conserved domains of AiGRF proteins. (**B**) The gene structures of *AiGRF* genes. Table S1: TPM values of *AhGRF* genes under combined drought and ABA treatment. Table S2: Molecular characterization of *GRF* genes identified in peanut. Table S3: Ka and Ks values for *AhGRF* genes. Table S4: Syntenic *GRF* gene relationships between Arabidopsis and *A. hypogaea*. Table S5: Syntenic *GRF* gene relationships between *A. hypogaea* and its diploid progenitors *A. duranensis* and *A. ipaensis*.
