# Supplementary material for: Genome-Wide Identification and Characterization of 14-3-3 Gene Family in Peanut
Source: Genes (Basel). 2026 Jul 30;17(8):901. doi: 10.3390/genes17080901 (PMC13512075; doi:10.3390/genes17080901)
Supplement: Supplementary file 1 [file genes-17-00901-s001.zip › Supplementary Figures.pdf]

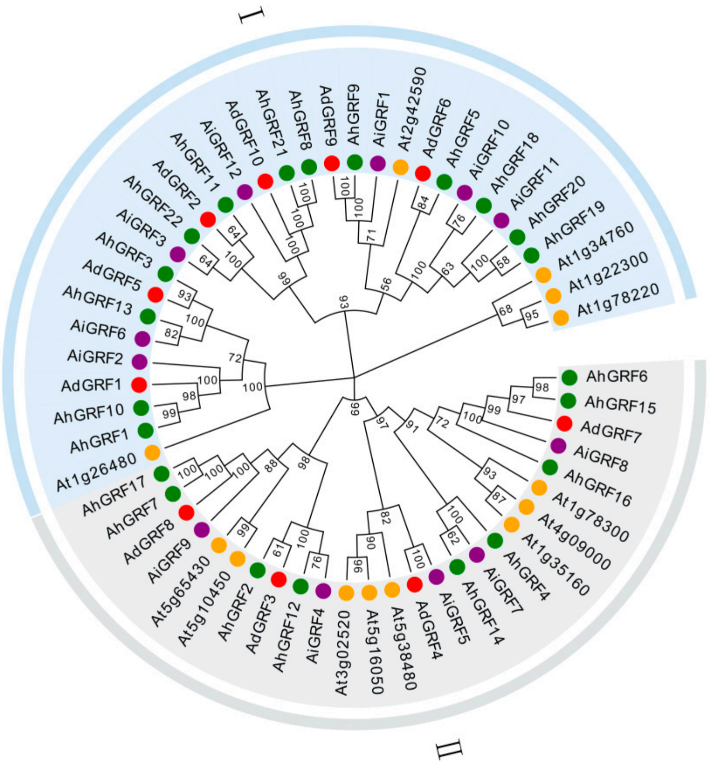

Figure S1. Neighbor-joining (NJ) phylogenetic tree of GRF proteins from Arabidopsis, *A. hypogaea*, *A. duranensis*, and *A. ipaensis*. Protein sequences were aligned using the Clustal W function in MEGA 11, and the tree was constructed with the NJ method based on 1,000 bootstrap replicates. Colors distinguish group I from group II. Green, red, purple, and yellow circles denote proteins from *A. hypogaea*, *A. duranensis*, *A. ipaensis*, and Arabidopsis, respectively. Bootstrap support values exceeding 50% are shown at the internal nodes. The resulting topology was visualized using Evolview. Phylogenetic analysis divided all GRFs into two groups: group I contained 31 members, and group II contained 26 members.

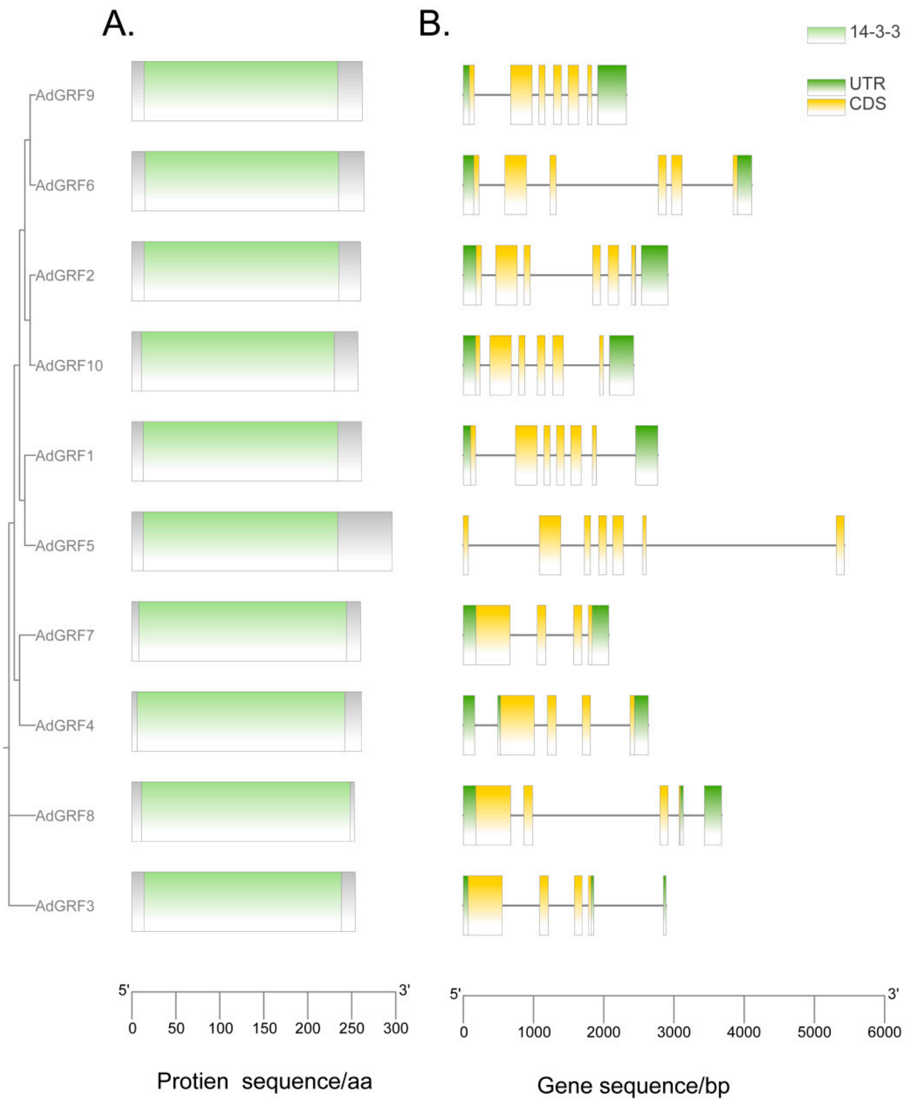

Figure S2. Structural features of *AdGRF* family in *A. duranensis*. (A) The conserved domains of AdGRF proteins. (B) The gene structures of *AdGRF* genes.

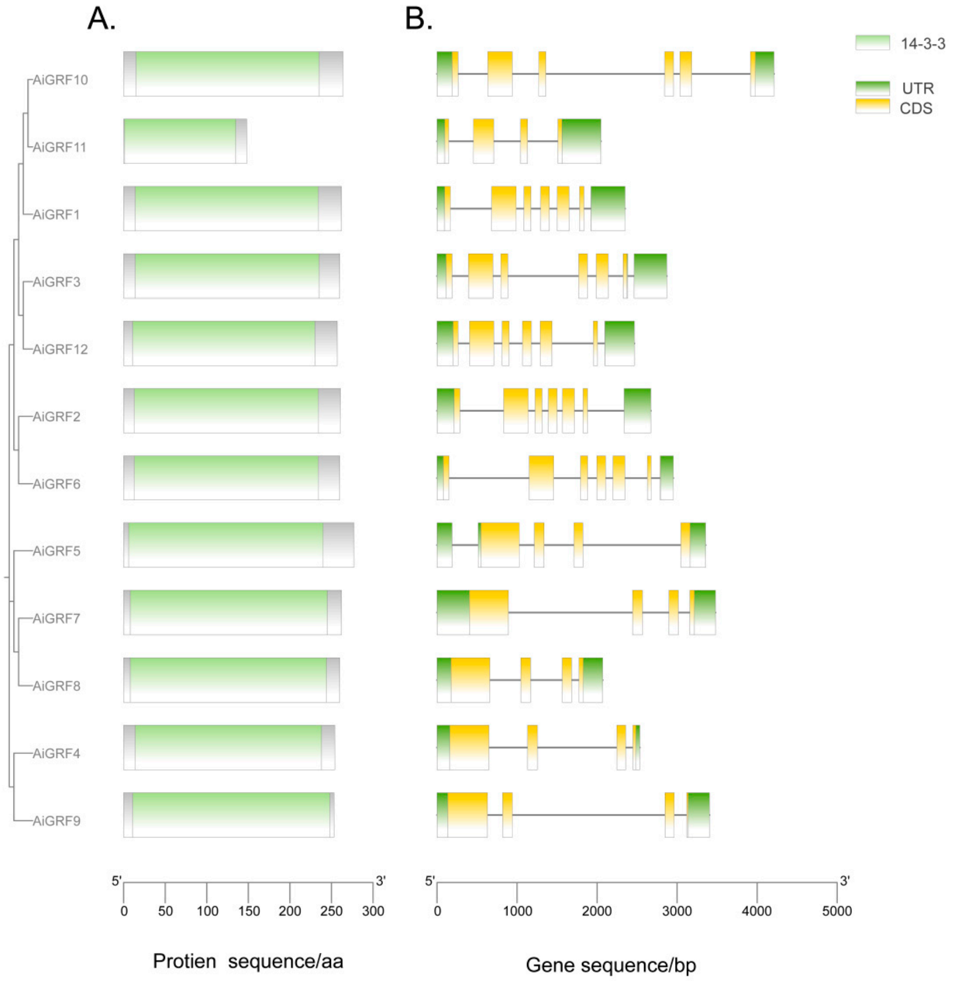

Figure S3. Structural features of *AiGRF* family in *A. ipaensis*. (A) The conserved domains of AiGRF proteins. (B) The gene structures of *AiGRF* genes.
